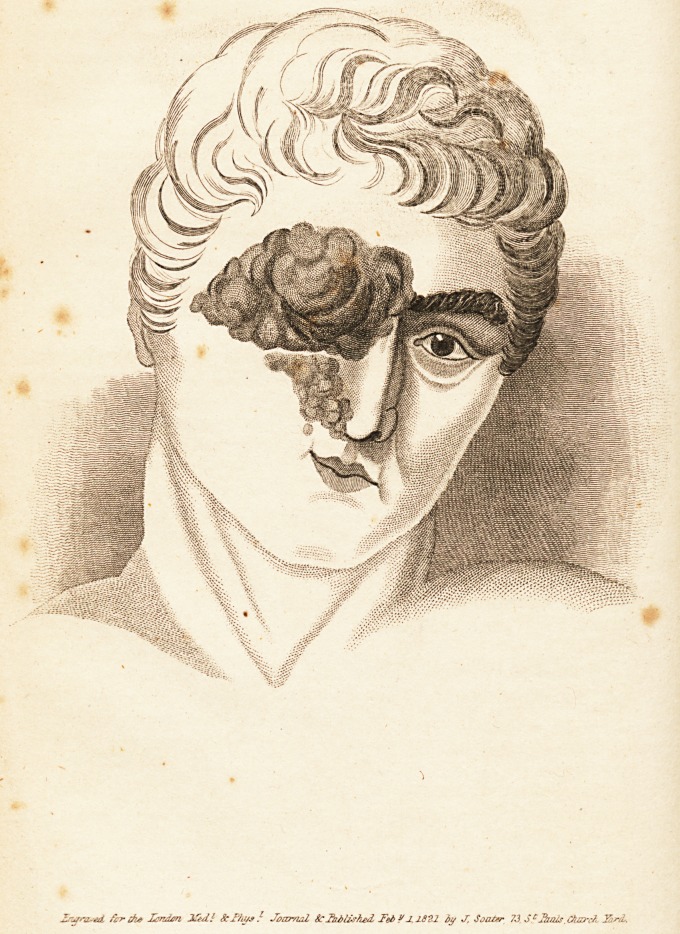# A Practical Treatise on the Diseases of the Eye

**Published:** 1821-02

**Authors:** 


### CRITICAL ANALYSES

OF

### RECENT PUBLICATIONS, IN THE DIFFERENT BRANCHES OF MEDICINE AND SURGERY.

"I would have men know, that, though I reprehend the easie passing over of the causes of things "by ascribing them to secret and hidden vertues and properties; (for this hath arrested and laid "asleepe all true enquiry and indications;) yet I doe not understand but that, in the practical "part of knowledge, much will be left to experience and probation, whereunto indication cannot "so fully reach: and this not only in *specie*, but in *individuo*. Yet it was well said, *Vere scire* "*esse per causas scire*."—BACON.

*A Practical Treatise on the Diseases of the Eye.* By JOHN VETCH, M.D. F.R.S.E. Member of the Royal Medical Society of Edinburgh, and of the Medico-Chirurgical Society of London; lately Physician to the Forces, and Principal Medical Officer to the Ophthalmia Military Hospital. 8vo. pp. 267; with three coloured Plates. Burgess and Hill. London, 1820.

WE know not a better exemplification of the advantages that have resulted from especial study of the diseases of a particular organ, subsequently to profound and comprehensive knowledge of the principles of general pathology, than that presented in this work; which is not more remarkable for the extent of the original observations comprised in it, than for the ability with which the author has every-where elucidated the facts he relates by his reflections, and the solidity of the general principles which he has drawn from them whenever the extent of existing knowledge would admit of deductions of this kind. With those merits, this treatise possesses the advantage of being constructed in a methodic manner. The whole of the author's descriptions, too, are traced with a degree of perspicuity and precision that is very remarkable, and cannot be excelled; and the impressions they make on the mind, in consequence of the possession of those qualities, are so strong and definite, that it is impossible for any person of ordinary intelligence to mistake or confound any thing which Dr. Vetch has stated. The same characteristics, in respect to strength and clearness, pervade the reasonings with which the descriptive part of the work is throughout accompanied. Altogether, we repeat, this treatise is a very extraordinary production. In endeavouring to show the correctness of those remarks, as well as such an abstract of the contents of the book as may be comprised in a few pages will permit, we shall, certainly, adduce many important observations which had been previously made known by others; but, in the greater proportion of these instances, it is clearly evident,—from the manner in which they are stated and illustrated by reflections and inferences peculiar to the author,—that, although they may have been original with the promulgators of them, they are, as far as regards himself, equally original with the author of this treatise. Although, therefore, by withholding so long from the profession generally, information of the results of his experience, Dr. Vetch has suffered others to anticipate him in the publication of several observations and points of doctrine, he will not lose the tribute due to him, from any one who will peruse this work with due attention; even were the evidence wanting that the greater part of the observations alluded to must have been made at periods prior to those of their publication in any literary record, and when, indeed, hardly any knowledge of the general pathology of the eye had been attained.

It is extremely gratifying, after having perused this work, to compare the knowledge we now possess of the diseases of the eye with that which was attained by English pathologists before the time of Mr. Sanders. A little empirical information of the appearances and consequences of such diseases, and a moderate degree of manual dexterity, constituted then all the qualifications of an *oculist*, and to such persons the treatment of affections of the eye was chiefly confided; for, though Pott and Cheselden, and some other men of eminent talents, had sudied them, their efforts were principally directed to the means of relieving them by operations: the consequences were, that, as Dr. Vetch has shown, we knew less of this subject than what was known by the ancients. There could not fail to be some apathy in practitioners in general in seeking for knowledge that would be of but little utility to them, and the deficiency of information was not likely to be supplied by the men who had confiscated to themselves the cure of such diseases.

This falling-off of the state of medical science in this as well as in other instances, commenced under the Arabians in Spain, when surgery was made a distinct department of the healing art; and when this again, in conformity with the custom of Asia and Egypt, was separated into many divisions, each of which was taken as the especial province of particular practitioners. In Germany, this subject has never been neglected by the generality of pathologists; and Italy has produced several excellent writers on it, subsequently to Professor Scarpa: but British ophthalmology has been founded in our own days, for Mr. Sanders was certainly the first (alluding to published observations,—not to the acquirements of individuals who, like Dr. Vetch, retained to themselves, for a time, their collected original knowledge,) who gave it a scientific character; and Mr. Wardrop, (Dr. Vetch remarks,) in applying to it the doctrines of modern pathology, especially those established by Pinel and Bichat, has certainly contributed much to render it an interesting study for the general practitioner in medicine: he must therefore be considered one of those to whom the success of this science is much indebted. There seems now to be no fear of this branch of the healing art falling again (whilst our present social establishments continue to exist,) into the hands of mere operators; and the respectable names already mentioned, with those of several other living practitioners, have raised the practice of it to its proper degree of dignity in the profession. The most gratifying consideration, however, is, that it has of late been so successfully cultivated, that there is, perhaps, no class of diseases better understood than those of the eye at the present day.

Adverting to the particular consideration of the work before us, we commence with stating that it is divided into two parts: in the first of which the author treats especially of the diseases which affect the eye itself; in the second, he considers those which are seated originally in the conjunctiva. The former division comprises some preliminary observations on the general Character and Treatment of Ophthalmic Inflammation, and treats particularly of ophthalmitis sclerotica,—ulceration of the cornea,—opaque cornea,—ophthalmitis iritica vel sclerotica interna,—lenticular and capsular inflammation,—and amaurosis: the latter, of ophthalmia, (which term is applied by the author only to inflammation of the conjunctiva,) arising from climate and atmospheric change,—catarrhal ophthalmia,—the purulent ophthalmia of the British army,—gonorrhœal ophthalmia,—the purulent ophthalmia of infants.

The author commences his remarks on the general character and treatment of ophthalmic inflammation, with what will, to many practitioners, appear a somewhat rash assertion: "Local inflammation," he says, "when it does not involve any of the parts immediately subservient to life, is a disease which we are able to command or subdue. Inflammation, more especially as it affects the eye, unaccompanied by constitutional disease, is always to be regarded as susceptible of being conducted to a successful termination:"—but, before such an appellation can be applied to it with propriety, it is necessary that instances should be adduced of the failure of the means proper for combatting it, when employed in the manner inculcated by the author. In the treatment of inflammation of the eye, he adds, we possess several peculiar advantages, from the singular opportunities we have of observing the phenomena, and ascertaining the precise limits of the disease, as well as from the uniform nature of the changes incident to its several stages; and hence a proportional degree of precision ought to be acquired in its management, especially in regard to the attainment of our object by the fittest method, and in adapting the extent of the means employed to the exigency of the case, as well as in regulating them according to their influence locally and on the system in general.

"In other instances of local inflammation," the author continues to remark, "the actual severity of the disease must of itself decide the extent or force of the treatment; but here, besides the degree of violence, the particular character which it assumes, according to the variety of structure which it occupies, must form an object of prominent consideration. The importance, therefore, which attaches to the difference of practice to be observed, for the cure of the inflammation of parts so very contiguous as those of which we are about to treat, and the characteristics of which I was early led to appreciate, by the strict evidence of facts, induces me to enter into a general view of ocular inflammation; believing, as I do, that the principles I am led to offer in explanation of practical results, will afford the basis of a more simple classification, and present such therapeutical indications as may prove no less conducive to the ease and decision of the practitioner, than to the security of the patient."

On proceeding to the particular discussion of those subjects, the author finds it necessary, in the first instance, to obviate the confusion which has been thrown around them by the indefinite use of the terms applied to them, that has been manifest in the writings of modern authors. Inflammation in two very distinct parts has of late been indiscriminately blended in the present acceptation of the word *ophthalmia*, which was originally employed by Hippocrates as a term for catarrhal or purulent inflammation of the conjunctiva; whilst the different forms of it were distinguished by the addition of specific or qualifying terms, to which the successive writers of the Greek, Roman, and Arabian schools implicitly adhered, but which have long ceased to convey any accurate or practical grounds of distinction. Dr. Vetch again restricts the use of the word *ophthalmia* to the sense above stated: inflammation affecting the eye itself, and cognizable as an external disease, he designates by the appellation of *sclerotic inflammation*, or *ophthalmitis sclerotica*, as the term which most distinctly describes the seat and nature of the disease. It might appear that *iritis* could not be properly comprised by this appellation, but the author, when treating of this affection, remarks, that, although inflammation may commence in the iris as a primary disorder, while the redness of the sclerotic coat appears to be secondary or symptomatic, "in either case, the formation of iritis is so blended with a modified inflammation of the sclerotic coat, that it is impossible to separate the consideration of the one from the other."

The following table will present a more particular view of the author's classification of diseases of the eye, which, he says, he has drawn up rather as an index to the observations he has to offer, than as an attempt to introduce a better arrangement of the subjects than may have been hitherto proposed.

"*Ophthalmia*, *or Conjunctival Inflammation.*

"Sp. I.—Catarrhal ophthalmia, or *ophthalmia mitior*, sporadic, endemic, and epidemic, with or without chemosis.

"II. Puriform, or *ophthalmia purulenta*, *ophthalmia gravior*, the *lippitudo*,* *ophthalmia vera*, *ophthalmia humida* of the ancients, *blephoroblenorrhœa*, and *ophthalmoblenorrhœa* of the German ophthalmologists.

"*a.* Ophthalmia of infants.

"*b.* ——————— produced by the infection of ophthalmic virus.

"*c.* ——————— by the infection of gonorrhœal virus.

"*d.* ——————— by the metastasis of gonorrhœal inflammation.

"*e.* Rheumatic, syphilitic, and arthritic.

"*Ophthalmitis sclerotica.*

"Sp. I.—Idiopathic, or corneal.

"II.—Iridial, or symptomatic."

The author—apparently deeply impressed with the idea that peculiarities in the *structure* of parts are essentially connected, as causes, with the peculiarities of the phenomena they develop in states of disease,—adduces, in the next place, an anatomical and physiological account of the membranes which constitute the globe of the eye; in which he especially considers the precise boundaries, distinctions, and vascular connections, of the parts in question, as well as exemplifies the influence of those circumstances on the phenomena of several diseases by some appropriate observations. The *cornea*, he remarks, although

* "The misapplication of *lippitudo* to a chronic and glandular disease is perhaps too inveterate to be now rectified; in its original sense, it applies to the acute stage of ophthalmia."

it differs from the proper sclerotica in its minute anatomy, is nevertheless a strict continuation of the latter membrane *as far as concerns its vascular connection;* which appears to be a fact of much importance, for, according to Dr. Vetch, "inflammation does not take place in the cornea, and consequently neither suppuration nor ulceration, until inflammatory action has been set up in the vessels of the proper sclerotica, even when the exciting cause is applied directly to the substance of the cornea. No symptom of re-action is visible, until that part of the sclerotic coat which is nearest the injured portion of the cornea has put on the appearance of inflammation: on the other hand, idiopathic inflammation once excited in the vessels of the sclerotic coat, invariably tends towards the cornea; and there, in consequence of the more destructible nature of the part, the common consequences of inflammation take place, such as effusion of lymph and ulceration." The very important practical indications which are founded on the above-mentioned facts, will be hereafter considered. The observations which ensue, on *iritis,* are derived from a further extension of the same views, and are still more interesting than those just cited: it is said that—

"The intimate connection formed on the internal surface of the coat with the iris and the ciliary structure, would naturally lead us to expect that the inflammation would have an early tendency to seize upon these parts: attentive observation, however, will prove that, in ordinary or idiopathic inflammation, any apparent affection of the iris is more the effect of sympathy than of the actual presence of disease, and that the supervention of iritis is, according to my view of the subject, to be considered as a distinct form of sclerotic inflammation, connected with some idiosyncracy, or morbid diathesis, previously existing in the constitution; and is, for the most part, more insidious in its progress than violent in its symptoms. As, before any degree of acute inflammation can establish itself either in the cornea or in the iris, a similar action has taken place in the sclerotic coat, so the farther progress of disease, in either of these parts, continues to be indicated by the greater or less activity of the inflammation in the sclerotic, and to this appearance too much attention cannot be paid. The feelings of the patient, the stationary appearance of other symptoms, may lull the practitioner into a fatal security, which he will best avoid by making the condition of the sclerotic coat the only safe test of the arrested progress of the disease."

In further support of those views, the author remarks, that injury done to the iris by accidental or artificial wounds excites no disturbance in the part or uneasiness to the patient, and will eventually heal without any troublesome symptoms, if inflammation does not appear in the sclerotic coat also; but in that case the structure of the iris is liable to be destroyed with all the signs of active and acute disease.

Some reflections ensue on the varieties in the character of inflammation dependant on diversities in the structure of the part affected: the violence, as well as the duration of it, the author says, "is, *cæteris paribus*, according to the resistance opposed to the distention of the vessels which have become the subject of inflammatory action;" and on this principle he endeavours to account for the longer duration of active inflammation in the sclerotica than in the conjunctiva. The ordinary course of observation is sufficient to convince us of the truth of the illustration, though we doubt the validity of the arguments adduced in support of the above proposition, as they all turn on the notion that the vessels concerned in acute inflammation are in a state of increased action, or, as the author expresses it, of *re-action* on the irritative cause.

There are some other diversities in the general characters of inflammation of the sclerotica and the conjunctiva which are worthy of attention, as the knowledge of them will aid in the establishment of the diagnosis of the two diseases. In the early stage of conjunctival ophthalmia, the inflammation is most observable at a distance from the cornea, round which the membrane often preserves, for some length of time, its natural appearance. Precisely the reverse takes place in the case of sclerotic inflammation, which invariably appears at the circumference of the cornea, forming a zone more or less complete around it. Intolerance of light invariably accompanies sclerotic inflammation, and is entirely unconnected with that of the conjunctiva. Having thus pointed out the more striking diversities in the character of the two affections just enumerated, the author runs through the inferior points in their history, describing them with perspicuity, and every-where producing important original observations. Chemosis—an elevation of the conjunctiva from effusion of fluid beneath it, dependant on the existing inflammation of that membrane,—he regards as a means of preventing, in the generality of cases where it takes place, inflammation of the conjunctiva extending to the sclerotica, which would otherwise ensue, from the contiguity of the two membranes.

Having discussed the more general laws of ophthalmic inflammation, the author enters into some considerations, of a similar kind, on the principles for its treatment. They may, he says, be comprised under three general heads: "the first, consisting in remedial efforts applied to the system, with the view of diminishing local action, of which venesection is the most certain and the most powerful. The second embraces the means of lessening the immediate impulse of the blood to the diseased part, by emptying the supplying trunks, or by giving a new direction to the existing impetus. The third consists in the application of agents to the immediate seat of the disease, by which the morbid state of the vessels may be altered or subdued."

Inflammation in the conjunctiva is but little affected by venesection, unless this be productive of syncope, which will prove the end or cure of the disease; whilst "the strength and fibre of the patient may be reduced, by abstinence and repeated blood-letting, to the lowest standard, without producing any material benefit, or insuring the organ against the destructive consequence of the farther progress of inflammation." But syncope, produced in the way just mentioned, has no such control over the inflammation which supports itself in the vessels of the sclerotica: in this case, however, the topical abstraction of blood, especially by cupping and leeches, has "sufficient control over the various states and individual symptoms to render any larger bleeding unnecessary." The application of leeches to the conjunctiva of the lower eye-lid, or to the septum nasi, is particularly advised. The exclusion of light from the inflamed eye, is, the author says, "often more detrimental than useful. The eye becomes more irritable, and less manageable when defended from the access of a moderate degree of light: its exclusion can only be permitted during the very early stage of inflammation. I have treated many thousand cases, and I have never suffered a shade to be worn."

"All washes, applied to the external parts of the eye, are for the most part worse than useless: the powerful agency of heat and cold ought to be employed with great precision and with due energy, or not at all. The eye-glass, or cup, used for the application of collyria to the surface of the eye, is troublesome and inefficient. The only proper mode of directing such applications, is by everting and extending the eye-lids, and injecting the fluid over the whole surface, which is best accomplished by an elastic gum syringe."

With many other experienced practitioners, the author considers that blisters near the inflamed eye too often increase the disease they were intended to remove: this is particularly the case with respect to the temples. There is but one exception to this statement as a general rule, which is, that blisters applied to the external surface of the palpebræ, in cases of purulent ophthalmia, tend considerably to diminish the purulency and chemosis.

"The argentum nitratum is a remedy which, in the hands of an oculist well skilled in the symptomatology of ocular inflammation, and capable of using it with the extreme delicacy necessary to insure its success, may often supply the absence of every other: the slightest application of it in substance can often remove the highest degree of morbid sensibility to light, and instantaneously restore quietude to the organ; it can prevent incipient changes, and obviate advanced ones, and may also be used in solution, as a valuable sedative.

"With respect to conjunctival ophthalmia, there is not, perhaps, another disease for which remedies of greater specific efficacy have been found. For the purpose of altering the violent and purulent state of the membrane, it is impossible to possess a medicine of greater efficacy than the liquor plumbi sub-acetatis, infused in an undiluted state. The effect of nicotiana, as a narcotic and astringent, applied externally, is of singular use in abating both the pain and the excessive tumefaction which attends the disease, and constitutes a very valuable addition to our other resources."

The most powerful and generally-useful internal remedies are belladonna and, more particularly, hyosciamus and stramonium; which, the author says, "the oculist can employ with a certainty more akin to the mensurable nature of physical force, than the uncertain operation of an animal stimulant."

The most arduous part of the cure of ophthalmic inflammation, results from the disease being but seldom confined to one eye: for, "from the sympathy, contiguity, and exposure of the two organs to the same causes, we have generally the same form of disease to combat in immediate succession." Here the author makes a remark, which is equally well applicable to many other cases,—that blood-letting so as to produce syncope, although so effectual a remedy for ophthalmia, will not, by any prophylactic power, prevent the disease which it will cure, and for which its repetition, on a new accession, will become necessary. The same may be said of mercury, when it exerts a specific efficacy in arresting inflammation.

Notwithstanding our attempt at the utmost conciseness, we find that an account of the author's general views occupies nearly half as much space as his own exposition of them; but, where almost every sentence comprises original observations of essential importance, it is hardly possible to give an abridged abstract. We could not well neglect to give such a comprehensive account of this part of the work; but we must pass over the particular disquisitions in a more rapid manner, and endeavour only to comprise in an abstract such points as will present something like a general view of the author's observations and doctrines, and recal to the mind of a person who has perused the work itself, the circumstances which are of most essential importance with respect to practice.

As the most serious and destructive effects of ophthalmia are owing to the extension of the inflammation to the sclerotica, the author thinks it convenient to commence his particular disquisitions with inflammation of the membrane just named. He first enumerates the several causes which may give rise to this disease, and which are generally pretty well understood; but there is a diversity in the original seat of it, in the opinion of the author, as it arises from external or local sources of irritation, and as it results from "some more general and pre-existing disease of the system," which has not been before remarked. In the former case, the inflammation occupies the external surface of the sclerotica, and is termed by Dr. Vetch *sclerotico-corneal inflammation;* in the latter, it has a tendency to proceed to the choroideal coat, when it is commonly known as *iritis:* this the author terms *sclerotico-choroideal inflammation.* The origin and progress of this affection was noticed in a former part of this article; but we may here repeat, that the author thinks it of great importance that it should be understood that, "whether the inflammation proceeds from the iris to the sclerotic coat, or *vice versa,* the inflammation has its active character and basis in the structure of the sclerotic coat." This form of the disease has a rheumatic character, without any definite termination, and may occur as the local manifestation of a rheumatic or arthritic diathesis. It is frequently met with as a symptom of syphilis; and apparently, also, as the effect of a mercurialized state of the system, where no syphilitic taint can be suspected.

In the first species of sclerotic inflammation—the *sclerotico-corneal,*—the first set of symptoms, the author says, may be considered as generic or common to both species, and may be comprehended under the following heads: "*an increased vascularity of the part, morbid sensibility to the impression of light, contraction of the pupil, pain, heat, augmented secretion of the lachrymal fluid, pyrexia.*" In his particular description of the phenomena of this affection, the author says that the encroachment of the vessels on the margin of the cornea,—as they radiate from the angles of the orbit to the centre of the eye,—and an immediate intolerance of light, are inseparable consequences. It is stated that the iris is seldom more safe from any actual attack of inflammation than when this action is proceeding with its utmost violence in the substance of the cornea; and that we never find the urgency of intolerance of light so distressing as in those cases where extensive destruction of the cornea is going on. The morbid sensibility to light keeps pace, too, with the visible inflammation of the sclerotica and the cornea, both in its advancement and decline. These are curious facts, and seem to confirm the opinion of the author, that it is on the morbid state of the cornea, rather than on that of the iris or retina, as commonly supposed, that this increased sensibility to light depends. The proposition is supported by several other, but less striking, arguments adduced by the author; and he remarks that—

"The relation which *photophobia* has to one part of the organ more than another, may at first appear of little importance: the hasty assumption, however, of its being referrable to the nervous or efficient structure of the organ, has led to two opposite errors in practice. An inflammatory or congestive state of the deeper-seated parts of the organ, so far from being accompanied by an augmented sensibility, more frequently occasions a contrary state; while, on the other hand, that weakness of sight, or inability to use the eyes in a strong light, often indicates an obscure inflammation in the sclerotic coat, affording two examples of inflammation attended by symptoms the reverse of each other; both require the employment of antiphlogistic remedies, and both are often aggravated by pursuing a contrary practice."*

The rest of the symptoms already enumerated as characteristics of this disease, are then individually considered, in successive order. The most remarkable circumstances respecting the *pain*, are its occurring in various parts of the head and orbit, and being subject to intermission and change of place. The matter secreted by the inflamed sclerotica does not partake of the essential qualities of pus, but preserves the tenacity and other properties of lymph. It seems to be an important point of knowledge in regard to the diagnosis of some affections of the eye, that "matter formed in the substance of the cornea never possesses fluidity." This tenacity of the matter, when it is thrown out on the external surface, is sometimes a source of the greatest distress and most severe pain with which the disease is accompanied, as we have experienced in our own person. The lymph, on becoming somewhat concreted, forms strong bands, adhering to the ulcerated surfaces, which, on being pulled by the eye-lids on every motion of these or of the eye itself, are the means of producing the most excruciating pains. This tenacity of the effused matter prevents its escaping from the little collections which form on the cornea, on these being opened into by a lancet. The protuberance formed by the collection is but slight, and the part thus affected either attains organization by the surrounding vessels ramifying into it, or is separated by ulceration.

The author's general precepts for the treatment of sclerotic inflammation, and some of the particular indications which arise from his particular views of the disease, have been already noticed. We can only add here, that he dwells strongly on the necessity of frequently-repeated local blood-letting, which is, in the generality of cases, the only mean of depletion of this kind that is requisite; though, he adds, "cases will often occur where it will be necessary to take away blood, and that largely, from the system also." Opening of the temporal artery has been strongly recommended, but, after trying it very

* "For the good effects attending the antiphlogistic treatment of this affection, see an Essay by Mr. Stevenson."

extensively, Dr. Vetch has been led to conclude that a smaller quantity of blood obtained by cupping, is of more real service than a larger quantity obtained in the former way. "The force of the general circulation may also be reduced by the exhibition of antimonials, given in such doses as will keep up a considerable degree of nausea, the direct effect of which in abating inflammation is great, while it produces the further good of forcing the consent of the patient to a due reduction of diet." Cold applications, in the way of wet compresses, and making a solution of opium or hyosciamus the means of applying the cold, are often beneficial. In some cases, the occasional use of fomentations, and the vapour of water and vinegar, produce most alleviation. But, the author adds,

"Warm poultices and long-continued fomentations are most especially conducive to the destructive consequences of ophthalmic inflammation,—the relief they may afford being treacherous in the highest possible degree; and so obvious is their tendency to effect relief, by accelerating the destruction of the cornea, that I should consider any patient entitled to recover damages, in whom the disease has terminated unfavourably, whenever it has done so under the application of a poultice."

The application of the argentum nitratum, formed into a finely-pointed pencil, to the vessels leading to the cornea, will be found to afford very great relief; and for this purpose it is sufficient barely to touch the surface of the conjunctiva with the greatest delicacy; after which the eye is to be immediately washed, by injecting a little tepid water with an elastic gum syringe. The following remarks, from so experienced a practitioner as Dr. Vetch, are particularly interesting; though the operation to which they relate does not appear to have been much employed by the generality of practitioners.

"When the pain is urgent, and the violence of the symptoms great, Mr. Wardrop has proposed the evacuation of the aqueous humour, by puncturing the cornea. The testimony which I have to offer on this subject goes more to establish the safety than the expediency of the operation. I have more than once had repeated recourse to the operation in the same eye, but at the same time the very necessity for its repetition has proved the effect to be less decisive than seems to warrant its adoption; and I have seldom ever performed it, where I might not with equal propriety have had recourse to it again. From the irritable state of the inflamed eye, it is often troublesome to accomplish; and in some cases, where the timidity of the patient has prevented the completion of the operation, instead of finding any bad consequences to ensue on relinquishing the attempt, the patient never failed to express as much sense of relief as if the instrument had actually penetrated the whole thickness of the cornea."

Some remarks on the use of hyosciamus and belladonna, as topical applications; on the beneficial effects of digitalis, internally, when intolerance of light remains, along with copious lachrymation and acceleration of the pulse; the necessity of a low diet; and the utility of purgatives occasionally, terminate the author's considerations on the treatment of this form of the disease. Until it has manifested an evident tendency to exhaust its action in the structure of the cornea, we cannot, he says, be too watchful of the state of the iris; on the slightest affection of which, along with the means of subduing the external inflammation, it will be necessary to combine those which insure the safety of that part. For this purpose, the extract of hyosciamus will be found an advantageous substitute for the belladonna, the effects of the latter being precarious whenever there is much inflammation of the proper structure of the eye.

*Ulceration of the cornea*, resulting from inflammation, is next considered. It may take place from a similar process having been set up in the conjunctiva, as well as from idiopathic inflammation of its own structure: in the latter case, it often commences by apparent apostemation in the central of the three laminæ of which the part is constituted; and it may terminate by proceeding inwards, so as to produce *hypopion*, or in an external ulcer. The author here adduces an illustration of a proposition which he had before stated: "The first general law regarding the morbid changes which take place in the substance of the cornea, is that, until the effect of an injury done to the cornea is propagated beyond its circumference, and until inflammation is there excited, it retains its natural sensibility. If no such inflammation takes place, the ulceration is conducted by the serous vessels, without producing any visible signs of inflammation to the observer, or pain to the patient." For Dr. Vetch's account of the various appearances, progress, and consequences, of ulceration of the cornea, we are obliged to refer the reader to the work: we can only notice here his explanation of the origin of *staphyloma*, which is new and satisfactory.

When ulceration has perforated the inner membrane of the cornea, the aqueous humour flows away externally, and continues to percolate until adhesion of the sides of the opening takes place or a small portion of new membrane is formed. If a second rupture does not soon destroy this reparation of the internal membrane, it quickly—that is, in a few hours,—projects through the external membrane, in the form of a pellucid and conical vesicle, the size of which increases in a corresponding ratio with the loss of substance which the ulcer may have occasioned in the middle and outer laminæ of the cornea. If the tumor enlarges very much, so as to occupy a considerable portion of the cornea, it is ordinarily termed staphyloma; and the same term, Dr. Vetch contends, should be given to its more limited formation, for in both cases the process is entirely the same. When staphyloma takes place in the centre of the cornea, and is so small as not to extend to the edge of the iris, the vesicle appears colourless from the first; but when the perforation of the cornea has happened within the limits of the iris, and immediate adhesion of that part with the inner circumference of the ulcer occurs,—constituting what is called *procidentia of the iris*,—the pupil is drawn to that direction, and the contact of the pigmentum nigrum gives a black hue to the point of adhesion, and communicates the same to the vesicle itself. As the vesicle increases in size, this black hue of it changes into a bluish tinge, whatever may be the colour of the iris. The resemblance which incipient staphyloma bears to the head of the house-fly, has obtained for it the name of *myocephalon.*

In the treatment of this affection of the cornea, it should be borne in mind that the ulcerative process can only be checked, when it is proceeding to destroy the inner membrane, by those measures which are capable of subduing the inflammation on which the action depends. When the immediate perforation of the inner membrane is threatened, we may with propriety resort to the operation of puncturing the cornea, at a place as remote as possible from the ulcer; "the operation being, I conceive," says the author, "fully warranted both by reason and experience." The indication of the ulcer healing is easily seen in the diminished activity of the inflammation, relief from pain, and the clear aspect of the part. The injection of vegetable tepid astringent infusions, or of milk and water only, may now be used. Next in importance to a diminution of the action on which the ulcer depends, is the removal of any slough formed either on its surface or in the adjoining part of the cornea. This separation may generally be effected by means of the point of a lancet or a couching-needle, round which the slough may be wound in such a manner as to enable the whole mass of it to be withdrawn at once. When it cannot be removed in this way, slight scarifications will cause it to be thrown off very rapidly. In using the instrument, it should be remembered that there probably remains nothing but the third tunic of the cornea to confine the aqueous humour. Sometimes, but always subordinate to the above indications, we may add some topical applications to the ulcer, as a solution of nitrate of silver, infusion of tobacco, or calomel applied with a camel's-hair pencil. The application of caustic to the incipient staphyloma, whether accompanied by procidentia of the iris or not, is another of the secondary measures. The author employs the caustic here in a mode very different from that recommended by SCARPA, who directs it to be applied until a slough is formed. Such an application Dr. Vetch considers would be fatal to the success of the remedy: it is quite sufficient that the caustic barely and instantaneously touches the surface. By this slight application, repeated daily, the incipient vesicle recedes, and the iris, though pointing forwards, is saved from any permanent adhesion. If the caustic touches, by accident, the edge of the ulcer, or any part but the apex of the projecting vesicle, it will often produce much mischief. The diversity in the modes of using this remedy inculcated by Scarpa and Dr. Vetch, appears to depend on the former considering the ulcer as the cause of the ophthalmia, and the latter the ophthalmia as the cause of the ulcer.

When staphyloma is of considerable size, there is much danger of its being burst by slight degrees of external violence; an accident which should, if possible, be avoided, as it is followed, generally, by excruciating pain.

The following observations will show the consequences of the more severe cases of this kind.

"Although the staphyloma when completed should extend over two-thirds of the pupil, that portion of pupil which remains behind the sound cornea suffers no kind of distortion, and is capable of a small degree of contraction and dilatation, though, for the cause I have already noticed, it does not serve the purpose of vision. The iris adheres to the tumor at each side, and that portion of it which remains beneath the sound cornea, whether it includes a portion of the pupil or not, takes the convexity of the natural cornea, but a sufficient quantity of aqueous fluid remains to prevent the consequences which would ensue from the actual contact of these parts: the fibres do not suffer that distortion which accompanies a more partial procidentia iridis, because, in the latter case, the iris is united to the centre of the part which the ulcer occupied; whereas, in a complete staphyloma, all within the base of the tumor is absorbed, a fact which I have had several opportunities of ascertaining by dissection. The only remains of the iris I found to be a few widely-separated striæ of pigment on the internal surface of the staphyloma. With a staphyloma occupying more than two-thirds of the pupil, it is surprising how seldom the lens is displaced by falling forward into the cavity of the staphyloma. The iris adhering at each side of the tumor seems sufficient to retain this body in its place, and, although the anterior chamber is wholly obliterated, the secretion of the aqueous humour is greater than in the natural state of the part."

The treatment of this affection has been regarded chiefly with a view to lessen the unsightliness of the tumor, for which purpose its removal, by cutting off a portion of it, has been recommended by Scarpa; whose own evidence, however, warns the reader of the severe inflammation and suppuration which are likely to ensue from the operation.

"There is, however, a much stronger objection to the operation," says Dr. Vetch, "in the fact, that an eye affected with staphyloma is not in so perfectly a hopeless state as it is but reasonable to suppose must be the case after such an operation as the one alluded to. Nature attempts the cure in every case, and in many brings it within the power of art to complete the success of her effort. While the process by which the thin membrane is converted into a white thick coriaceous substance is going on, the repeated rupturing of the parts which have not yet suffered this conversion diminishes the tumor, which, finally, contracts and subsides to the natural shape of the cornea, leaving an indelible leucoma over the place which it occupied. An artificial pupil may then be formed with the same favourable prospect of success as in any other case of obliteration of the pupil from leucoma of the cornea.

"The evacuation of this humour is often required to relieve the sudden and violent attacks of pain which its distention excites in the eye, or in distant parts of the head. Though I never measured the quantity of water which escapes, I think, in some cases, it must have amounted to two drachms. The incision heals immediately, and in a few hours the water is re-produced. I object, therefore, both to the removal of the apex of the tumor, as recommended by Scarpa, as painful and hazardous, besides being destructive of all chance of recovery; and to the puncturing of the tumor, as an endless operation, from which no permanent effect takes place. I have had recourse, with success, to caustic, and to the introduction of a seton in order to accomplish the gradual diminution of the tumor, and to bring the eye into that state where an artificial pupil may be made: at all events, to destroy the deformity of the projection, without the risk of a severe attack of inflammation and suppuration of the eye-ball."

The cornea is liable to a very considerable alteration of its shape, without any evident change of its structure, by which it assumes the form of a cone. "In all my attempts to restore the natural convexity of the part," says the author, "I have failed: evacuating the aqueous humour, removing the lens, and the application of long-continued pressure, have not been attended, as far as I know, with any improvement of vision."

Sometimes the cornea suffers a loss of substance, by a process of absorption different from that which takes place when that action is combined with the more obvious signs of inflammation; the result of which is a pellucid dimple. It seems to depend on a removal of the middle lamina of the cornea. It is not productive of much inconvenience; and, when formed, often remains stationary for a very long period.

*Opacity of the cornea*, consequent on primary conjunctival inflammation, is next treated of. The surface of the eye sometimes presents, from the cause just designated, a rough or granulated appearance, with a varicose state of the external vessels and a secretion of puriform matter; and universal opacity of the cornea takes place from the irritation produced by the disease of the conjunctiva. The lining of the palpebræ, especially that of the upper, partakes of the disease, and presents a remarkably villous appearance in the first stage of it, which is succeeded in process of time by deeply-sulcated, hard, or warty, granulations. We must refer the reader to the work itself for a more particular history of this affection.

"In the whole class of diseases of the eye," says the author, "and its appendages, there is no one affection so distinctly pointed out, and its treatment so uniformly described, by all ancient authors, as this state of the linings of the palpebræ, termed by the Greek writers* *trachoma* and *sycosis*, from the granular appearance of the surface; by the Latins, *scabies* and *scabrities palpebrarum;* and by the Arabians, *sebel.*†

"So completely, however, had such knowledge been overlooked in this country, that the real nature, as well as the cure, of the complaint may be considered as a recent acquisition; and we have seen the most unworthy claims set up as to the merit of introducing both the one and the other. The use of actual cautery, excision, and friction, for the purposes of curing the diseased state of the eye-lids, may be traced back to the writings of Hippocrates, and these measures seem to have been employed both separately and combined. The destruction of the granulations by friction, appears to have been the practice more generally followed in former times. The adoption of the more preferable mode of practice, by the application of escharotic agents, may, I think, with justice be conceded to Mons. De St. Ives: this author, whom I have not consulted till lately with that attention to which he is so well entitled, appears, in his own country, and at the distance of exactly one hundred years, to have performed a similar service to surgery, which, in our time, has been conferred upon it by the late Mr. Saunders, whose high qualifications and talents have now so successfully established the cultivation of ophthalmological science among us."

Dr. Vetch is decidedly adverse to the treatment of this affection by excision, preferring, as above shown, the application of escharotics. "After giving a fair trial to a great variety of this class of medicines," he says, "applied to the surface of the upper eye-lid in the form of ointment, I was led to trust to their application in substance alone, by which the treatment became so decidedly successful as to enable me to calculate with cer-

* Trachoma asperitas intra palpebram, est hujus intentio, ut velut incisuras habeat sycosis, a ficus similitudine vocatur, ubi vero diu duravit, et callum contraxit tyosis, id est callositas nominatur, si non his cadet inversam palpebram per pumicem rademus aut per sepiæ testam, aut fici folia, aut etiam per instrumentum *blexharoxyston* ab hac opera appellatum.

† Quum palpebra inversa est interius apparet rubra et aspera, et scabies adest, et cum super alba oculorum et super nigredinem, videtur similitudo panniculi ex venis rubris et crassis texti adest passio quæ vocatur *sebel.*—*Rhases in Regem Almanzorem.*

tainty on the cure of the disease, when not opposed by moral obstacles which no means could control." But, notwithstanding the evidence adduced by the author, as long since as the year 1806, of the superior merit of this mode of treatment, that by excision has been established in the practice of the army: "but," Dr. Vetch asserts, "it never could have received the trial which has been given to it, if left to the unbiassed decision of the profession; and, when time shall have removed all personal consideration, the mode of practice which succeeded with me will maintain its reputation, when that by excision will be left without an adherent." The following recapitulation will show the grounds for the foregoing decision, at the same time that it will point out, more particularly than we have yet done, the mode of employing the means of cure here recommended.

"First, that, of itself, the operation, however frequently repeated, is unequal to the cure of opaque cornea; while, on the other hand, the treatment I adopted in the disease does not require the aid of an operation in one case out of fifty.

"Secondly, that the operation, besides being in itself very painful, requires to be indefinitely repeated, and is often followed by inflammation; while the treatment by the properly-graduated application of caustic substances produces neither pain nor inflammation.

"Thirdly, in many cases where a new and white surface has been obtained after the repeated use of excision, the cornea often remains vascular; a circumstance which never happens when the cure of the membrane lining the eye-lid has been effected by the action of escharotics, properly applied, the cure of the cornea invariably keeping pace with that of the membrane.*

"Lastly, the claim of Sir W. Adams to an improved method of treating this disease, as announced in a circular letter of the Right Hon. the Secretary at War, is limited to the use of a knife in the place of the scissors, as employed by the late Mr. Saunders: to this novelty, if such it can be called, the objections now urged apply with tenfold force.

"The connection of the disease of the cornea with that of the lids, and the eversion of the latter for treatment or examination, was practised by Mr. Saunders, who taught it to Sir W. Adams, and was resorted to by myself, without any knowledge of the practice of Mr. Saunders or the existence of Sir W. Adams; though I did not then, nor do I at this moment, consider the eversion of the eye-lid to be generally necessary to the cure of the disease: its frequent repetition has appeared to me not only unnecessary but prejudicial; and I have met with more success by simply raising it with the thumb of the left hand, so as to admit the application of a small porte-crayon armed with the blue-stone or nitrate of silver, than I ever did by the com-

* Vide Observations relative to the Treatment of Ophthalmic Cases of the Army.

plete eversion of the palpebræ; nor will I yield the evidence of my own sense and experience on this point to any conjectural reasoning whatever. The treatment of this affection forms but one feature of a disease which, at the time that I met with it, was new to the profession in this country; and, although the practice proved successful to the utmost possible degree, it has been condemned without inquiry and without appeal, and in a quarter where professional discussion cannot be entertained.

"The whole power of high and official patronage has been employed to ensure the success of the operation for removing the granulation of the palpebræ by the knife; the failures have been concealed by every possible subterfuge. Nature, however, has proved her superiority; and it is now generally known that the operation, while it has been highly injurious in some cases, has only proved successful when aided by those applications which it was introduced to supersede; and this success has been so limited, as to prove that their use is not yet properly understood where the greatest pretensions have been advanced."

It will sometimes be advisable to take blood from the temples by cupping, to remove increased action of the vessels of the sclerotic coat, (one of the indications in the treatment,) at the same time that the measures are employed for the cure of the affection of the palpebral linings. The escharotics, for the latter purpose, should be pointed in the form of a pencil, and fixed in a port-crayon, as already mentioned; and they are to be applied, not, as some have conceived, so as to produce a slough over the whole surface, but with great delicacy, and in so many points only as will produce a gradual change in the condition and disposition of the part. It is not necessary at all times that the escharotic should be applied to every part of the diseased surface, for, when a healthy action re-commences, it soon becomes general. As long as any purulency remains, the above applications will be much aided by the daily use of the undiluted *liquor plumbi acetatis*, applied by means of a camel's-hair pencil. When the disease resists these remedies, and the surface becomes hard and warty, the author has had recourse to finely levigated verdigris or burnt alum, and sometimes the kali purum, applied in the way just mentioned; taking care that they are washed off by means of an elastic-gum syringe before the eye lid is reverted. All the substances above enumerated, when used in the way described, are to be here regarded, the author says, as acting, not as escharotics, but as astringents. Some practitioners have attempted to aid their efficacy by astringent washes; but these are always injurious, as they act also on parts which should be preserved from their irritative agency. The solid substances can be applied with precision to the points only on which it is desirable that their influence should be exerted. The liquor plumbi acetatis was usually employed by the author in the worst stage of the disease, and was changed, as the cure proceeded, for a solution of alum, applied by a camel's-hair pencil.

The excision of a portion of the conjunctiva immediately surrounding the cornea, recommended by Scarpa, was employed in the treatment of the cases in the military hospital, under the direction of Dr. Vetch, so as to give it a fair trial; but, it is said, without any good effect, excepting in cases of great relaxation of the membrane covering the eye.

The division or excision of the large varicose vessels which usually unite towards the external angle of the eye, has little or no effect, as new vessels immediately appear in the room of those removed by the operation, and the discharge of their contents does not compensate for the excitement which their excision occasions.

The prognosis in this disease is always favourable; and the more completely the cornea has become soft and opake, the more easily it is cured; though, if the patient's age is much above twenty, he is for some time liable to a relapse.

The fundamental points of the author's pathology of *ophthalmitis iritica*, or, as it has been commonly termed, *iritis*, have been already stated in this article, and we could add but little to what we have adduced on this subject without entering into details for which it is more proper that the reader should be referred to the work. Many observations are here brought forward in confirmation of the author's proposition respecting the insensibility of the iris, even when suffering inflammation. "Iritis," he says, "when it takes place, which it so often does, without any external exciting cause, may, unless sclerotic inflammation comes on also, eventually destroy vision, by the progress of adhesive action, without giving the patient any further warning than what arises out of the gradual loss of function. The instant, however, that the sclerotic coat reddens, the progress of the primary affection is accelerated and accompanied by the usual signs of acute disease." When speaking of the rheumatic character of iritis, Dr. Vetch remarks that "the resemblance which the structure of the sclerotic coat bears to that of tendinous expansion, necessarily disposes it to take on the same form of diseased action to which such parts are subject. The translation of rheumatism and arthritic inflammation to this part is, therefore, consistent with the habitual tendencies of both diseases." The author here, as well as in several other parts of the work, evinces a decided concurrence with PINEL and BICHAT in their views of the influence of structure on the character of inflammation: in conformity with which it may be said, that the peculiar characters of rheumatic inflammation depend on the inflammation having its seat in a fibrous membrane.

Dr. Vetch is not perfectly satisfied with the explanations of either Dr. Farre or Mr. Travers of the curious circumstance that mercury, although it seems to act as a cause in the production of this disease, is nevertheless one of the most efficacious remedies for it when established. He offers himself an hypothesis of the relations of those supposed facts, which is founded on the opinion that inflammation immediately results from the absorbent veins not carrying forward the blood with an activity proportionate to that by which it is brought to them by the arterial capillaries. Mercury excites the action of the absorbent veins, and thus produces a due relation in those functions, which is consequently followed by the disappearance of the inflammation. In endeavouring to account for the production of the inflammation by mercury, he says, "in proportion as the functions are excited beyond their natural standard, any sudden check given to the mercurial influence will necessarily interrupt the balance which it has established in the state of the circulation, raised at the same time beyond the natural standard. According as the operation of the interrupting cause is general or local, the effect will be a state of general fever or topical inflammation."

Dr. Vetch, however, in admitting the influence of mercury in the production of iritis, does not seem inclined to regard its operation as direct: he appears to consider it as acting only by "intermediate relations," as Mr. Pring would express it; and, viewing the subject as it is illustrated by the doctrines of causation of this physiologist, there is not the least difficulty in conceiving how the disease may be both produced and cured by means of mercury.*

On treating of the sensible appearances of *ophthalmitis sclerotica interna*, which are in general similar to those of the external sclerotic inflammation, and manifest the same zone of inflamed vessels surrounding the cornea, Dr. Vetch says, that a more leaden hue of the surface of the sclerotic coat, and the want of disposition to encroach upon the cornea, chiefly indicate the more internal course of the disease. The intolerance of light is less troublesome than in corneal inflammation, and,

* The following corrollary remarks, although chiefly interesting in another point of view, relate too intimately to the etiology of iritis, to permit us to pass them over here. Dr. Vetch says, "That the use of mercury materially aids the absorption of venereal virus into the system, when it would otherwise produce only a local disease, is now admitted: when given at all, it should therefore be persevered in until it has enabled the system again to expel the virus which it has absorbed. The small quantity of mercury given by some practitioners to secure the system, as they say, is, no doubt, the fertile cause of constitutional symptoms."

as the disease extends itself more and more to the iris, this symptom, instead of increasing, sensibly diminishes. Flashes of light are often supposed to be seen, when the irritation of the iris is great; for the same reason, that noises are perceived in the ear, when the organ of hearing is irritated.

In the worst cases of this disease, besides the lesions already mentioned, such a change in the structure of the sclerotic coat is liable to happen, that it can no longer preserve its regular spherical form, but suffers a partial projection in some part more than another, known by the name of *staphyloma sclerotica.* Hypopion, cataract, and amaurosis, are other occasional consequences of sclerotic iritis; the whole of which, as well as the more ordinary progress and consequences of this disease, are minutely and perspicuously described by the author, with all the originality of manner which marks the rest of the work.

The treatment of this affection should not materially vary from that proper for ophthalmitis externa, except in the use of mercury and belladonna, or hyosciamus, from the commencement; and, from the importance of the organ here affected, in the extreme vigilance and activity necessary in the employment of local blood-letting. "A small quantity of mercurial ointment with opium rubbed into the eye-lid and temple, night and morning, seems," Dr. Vetch says, "to have a specific agency in arresting the progress of the disease in the iris. The same may be said of the local use of stramonium and hyosciamus, which not only prevent the contraction of the pupil, but, after the removal of inflammation, appear very evidently to increase the mobility of the iris, by disengaging it from the adhesions it has formed with the capsule of the lens."

Dr. Vetch concurs with former experienced writers, in stating that, although the disease has occurred during a course of mercury, we may renew the exhibition of this medicine with safety and advantage. The local application of it, in the way just designated, will be sufficient when the iritis is not connected with syphilis, in which case it should be employed in a more effective manner: the rest of the treatment is not to vary from that which is proper when the disease arises from other causes. The recurrence or increase of pain, it seems important to remark, is always regarded by the author as an indisputable and unequivocal symptom of augmented inflammation.

*Lenticular and capsular inflammation*, and its consequence, *cataract*, are next treated on. Besides the inflammation from which cataract most frequently originates, the lens is subject to a more acute and destructive form of inflammation, which, in contra-distinction to that producing the more gradual loss of transparency terminating in the lenticular and capsular cataract, may be termed *lentitis.* Cataract exemplifies the adhesive process of inflammation,—this the suppurative. When suppuration is established, ulceration of the cornea comes on in process of time, and the aqueous humor is evacuated. The author seems to regard the diagnostic remarks of cataract and amaurosis proposed by Professor Beer as the most accurate. Professor Beer says,

"First, in cataract, all objects, especially white ones, appear involved in a thin cloud or mist; second, the vision diminishes in proportion to the visible cloudiness behind the pupil; third, the cloudiness shows itself most distinctly towards the centre, seldom at the border of the pupil; fourth, as the cataract increases, a blackish ring is observable at the border of the pupil, especially in light-coloured eyes; fifth, at first cataract obstructs the vision of objects directly opposite to the eye, but, when viewed sideways and in a moderate light, they are discerned with tolerable clearness; sixth, dioptric glasses aid the vision of cataract patients, so long as the cloudliness behind the pupil is inconsiderable: the flame of a candle appears to an eye in which cataract is forming, to be surrounded by a whitish circle or vapour, which appears broader the farther the patient removes from the light: if the cataract be completely formed, the patient can no longer see the flame, and can merely say where it is. Lastly, incipient cataract does not influence the mobility of the iris; if, at last, its movements are impaired, the complaint is by that time sufficiently obvious. On the other hand, the appearances which characterize the formation of amaurosis, are the great depth at which the cloudiness appears behind the pupil on looking at the eye sideways; from the cloudiness appearing somewhat concave; from its colour being more of a greenish or reddish hue. The diminution of vision bears no proportion to any perceptible cloudiness; the pupil is more or less expanded, the iris little or not at all movable; the pupillary border angular, and the pupil not perfectly circular; the cornea loses its natural and healthy aspect. In incipient amaurosis, there is also a remarkable increase or diminution of vision, not affected, as in cataract, by the degree of light or expansion of the pupil, but depending on physical and moral causes, affecting the sensibility of the individual: violent emotions of mind often giving a temporary increase of vision; while it is evidently diminished by long fasting, restless nights, great anguish, sudden fright, or excessive venery: under the operation of such causes, incipient amaurosis often terminates in permanent blindness. To the amaurotic patient, the flame of a candle appears as if involved in a mist, but, unlike the white cloud already described, exhibits, as well as the flame itself, the colours of a rainbow. Glasses are of no use to the amaurotic patient, and he distinguishes objects at the side with as much difficulty as those directly opposite to the eye."

All the species and complications of cataract are then considered; for an account of which it is absolutely necessary that we refer to the work. The general principles for the treatment of the two kinds of cataract, independent of the operations for them, must be manifest on the slightest consideration on the pathology of those affections: the greatest difficulty generally consists in determining the influence of local or of constitutional exciting causes. To these remarks we may add the citation of the following paragraph:

"Practitioners, who give themselves little trouble in investigating the particular nature of cataract, too often satisfy themselves and their patients by telling them to do nothing, but wait till the cataract is ripe or fully formed,—that is, until they are deprived of all useful vision. It is obvious, however, that in all cases depending upon inflammation, however slow and however obscure, it is the duty of the practitioner to prevent the further formation of the disease, by the means directed to subdue such diseased action; and, generally speaking, the same line of treatment which has been recommended for iritis, is equally applicable to the early stage of capsular inflammation; and it will be greatly aided by the use of setons or issues and rubefacients."

*Amaurosis* is the next subject of the author's observations, and it furnishes him with an occasion for adducing some forcible illustrations of a pathological principle, apparently true, which he seems particularly desirous to establish, and the knowledge of which is of great importance in regard to practice. He had already remarked, on speaking of iritis, that inflammation of the nerves of the senses properly is not accompanied with increased sensibility, as is the case with the other nerves; but, on the contrary, with a diminution of their proper sensibility: hence, inflammation, or increased vascularity of the retina, instead of being attended with increased power of perception of light, is productive of amaurosis. It is common to hear this affection spoken of as a state of *debility*, or want of excitement of the optic nerve, and not of inflammation, on the analogy that irritation of nerves in general is accompanied with increased sensibility; but, Dr. Vetch remarks, it will be found that the causes of amaurosis are often those which are productive of increased determination of blood to the head, and to the eyes especially; and he, by a series of arguments, shows that amaurotic blindness may be the consequence of direct inflammation of the retina, or of the vascular structure on which it rests. But, whilst he establishes this point, he takes care also to point out that it may in some cases arise from "local debility influenced by repletion of the system, obstructed circulation," and from "actual loss of power, from the natural decay of age or protracted debility." He however considers that, excepting when it occurs at a very advanced period of life, it is almost always formed out of some pre-existing disease. After the preliminary pathological reflections above designated, the author gives a very perspicuous analysis of the symptoms, with an account of the disordered sensual phenomena, and a description of the various appearances of the eye, which accompany this affection: he then passes in review its causes in a more particular manner, and concludes the chapter with a few remarks on its treatment. This, as his pathology indicates, should in many cases consist in antiphlogistic measures; and blood-letting, productive of syncope, will often be necessary to subdue the inflammation of the retina, or of its subjacent membrane, on which it depends. Purgatives, antimonial emetics, pediluvia, seclusion from the light, cold applications to the eyes, blisters and rubefacients to the neck, and the like measures, should be employed according to the indications present.

"It will be long," says the author, "before the utmost success of a rational treatment can compensate for the cases which have been rendered irremediable by the empyrical use of stimulants, and more especially of galvanism and electricity. These even at the present day are resorted to, and recommended without any reference to, or discrimination of, the causes to which the disease is owing. What can be supposed more mischievous than the application of electricity to an amaurotic eye, when it is perhaps occasioned by some organic disease, and thus stimulating a part already suffering from over-excitement. Without first removing the primary affection; by a stimulating plan of treatment, we not only take away all power of recovery from the part, but very likely bring on a state of actual suffering in addition to blindness for life."

When it proceeds from derangement of the stomach, Dr. Vetch recommends antimonials in small doses, which he has combined with the arnica montana. Richter is known to have been particularly successful in the treatment of this affection: his chief remedy was an emetic, repeated once or twice a-week; and this mode of treatment, with tartar-emetic administered to the extent of only one grain daily, on the intermediate days, has of late acquired much reputation on the continent.

We now arrive at the second part of the work, which is on inflammation of the conjunctiva. *Catarrhal ophthalmia*, arising from climate and atmospheric changes, which the author says are the most frequent exciting causes of ophthalmia, is the first subject for illustration. Here, as on former occasions, Dr. Vetch commences with some preliminary observations and reflections on the general pathological relations of the peculiar structure of the part which is to become the subject of his especial considerations; and thus, after the manner of Pinel in his Nosography, he prepares the reader for the ready comprehension of the particular details into which he subsequently enters. After pointing out, with accuracy and conciseness, adducing here and there original observations, the most remarkable circumstances in the pathology of mucous membranes, and showing their especial liability to become diseased from atmospheric influence, the author adverts to the ophthalmia of Egypt,—a country which, of all others, seems to be the most favourable to the production of inflammation of the conjunctiva; and the causes of which he, in the first place, endeavours to determine. He shows, satisfactorily, that it is not, as it has commonly been supposed, on the sands, or the influence of the drying winds, that it depends; but on the extreme humidity and relative coldness of the atmosphere during the night. An opinion very early published by Dr. Vetch, in the fourth volume of the Edinburgh Medical and Surgical Journal, and in which ASSALINI and Mr. POWER coincide with him; though each of them seems to have been led to it, nearly at the same time, by their own observations.

Some remarks ensue on the catarrhal ophthalmia of Europe. The author applies the term *catarrhal* rather than *purulent* to the ordinary ophthalmia of temperate climates from atmospheric influence, although the matter formed "may, and does," he says, "generally assume more the properties of pus than mucus;" but he chooses to reserve the term purulent for the more severe form of ophthalmia, which occurs from inoculation or infection. He considers, and apparently on good grounds, that ophthalmia, like fever, may first arise from atmospheric influence, and then afterwards be propagated by contagion, by the matter formed being communicated from the eyes of the diseased to those of the healthy; a proposition which he has satisfactorily and originally established in regard to the ophthalmia acquired by the British army in Egypt, in the commencement of the present century, and afterwards propagated in several parts of England and Ireland, when the soldiers affected with it were dispersed (a very interesting account of which, full of important information and suggestions for military officers and army medical practitioners, is given by the author,) on the return of the army. Dr. Vetch, indeed, goes so far as to advance as a general proposition, that, "from whatever cause inflammation of the conjunctiva may originate, when the action is of that nature or degree of violence as to produce a puriform discharge, the discharge so produced operates as an animal virus, when applied to the conjunctiva of a healthy eye." If this proposition might be considered as well established, (and the accuracy of observation, experience, and solidity of judgment, of Dr. Vetch, must produce much aversion to doubt its truth,) what a fertile source of reflection and indications for original pathological researches would be here presented to us. There is no obvious reason why other mucous membranes, the urethra for instance, should not be subject to similar laws; and, if this be the case, many hitherto anomalies and perplexing circumstances in the history and propagation of gonorrhœa might be readily accounted for. A little speculation will present a multitude of circumstances relating to fever and several cutaneous diseases, that seem to render it probable; but, in the present state of our knowledge, further observations and experiments should precede theory on this subject.

A very curious fact respecting this ophthalmia, analogous to that for which Mr. Jesse Foote first argued in regard to gonorrhœa, which Dr. Vetch has ascertained, is that the purulent matter from the eye of one man has the power of infecting the urethra of another man, and of producing gonorrhœa; whilst it is innoxious to his own urethra. This law the author has never known to be violated. This subject is more extensively discussed in the chapter on *gonorrheal ophthalmia*, and the discussion necessarily comprises some reflections on the laws of metastasis of diseased action; because it has been made a question in respect to gonorrhœal ophthalmia, whether the ophthalmia arises from *metastasis*, (or, as it is with more propriety termed by Mr. Pring, *related extension of disease*,) or from the application of the gonorrhœal virus of the patient, or of another person, to the eye which has become affected.

If ophthalmia be the result of the application of gonorrhœal matter of the same person to the affected eye, it is obvious that the law above stipulated for is violated; and ophthalmia coexisting with gonorrhœa would appear to be too frequent an occurrence to permit the supposition that it has arisen from the contact of gonorrhœal matter of another person. Although the attempt to excite ophthalmia by the application of gonorrhœal virus is too hazardous and imprudent an experiment, because of the dangerous consequences that might result from it, the converse of this—when the great importance of the question in regard to military discipline, and the fact that soldiers have commonly wilfully attempted to produce diseases, (even the Egyptian ophthalmia,) are considered,—may (when the consent of the parties is obtained, and when it was thought to be not improbable that, if gonorrhœa were excited, it might relieve the disease of the eye,) be considered justifiable. Dr. Vetch, therefore, applied the matter formed by the eye in ophthalmia to the urethra in several instances, but no disease ever resulted from it when the inoculation was effected in the subject from which the matter was taken; whilst, in one case, (the only experiment, it appears, of this kind,) the ophthalmic matter produced very severe gonorrhœa in another individual than that from whom it was taken.

From the results of these experiments, Dr. Vetch was led to regard the converse view of the subject as a fact: he says,

"From the result of these cases, I could no longer admit the possibility of infection being conveyed from the eyes to the gonorrhœal discharge of the same person. Some time after this the improbability, or rather impossibility, of this effect, was rendered decisive by an hospital assistant, who, with more faith than prudence, conveyed the matter of a gonorrhœa to his eyes, without any affection of the conjunctiva being the consequence. From this time I was led to look for an explanation of the connection subsisting between gonorrhœa and ophthalmia, arising in the same person, in some peculiarity of the constitution; and to conclude that the disease is an extension of an inflammation which first showed itself in the urethra, and of which the different structures of the eye are liable to participate, in common with many other parts."

It happens, unfortunately, that the author has not made it evident whether the experiment of the hospital assistant was made with the matter of a gonorrhœa affecting himself or another person; but we must, with almost absolute confidence, suppose it were from gonorrhœa affecting himself, (from the nature of the context, and Dr. Vetch in another place shows that ophthalmia may be produced by the contact of gonorrhœal matter;) and, if so, it might appear that the conclusion immediately following the account of it, is not quite satisfactorily established, because the gonorrhœal ophthalmia ordinarily witnessed may arise from the matter of another individual coming in contact with the eye of the patient, and probably at the same time that the gonorrhœal infection has been received. Dr. Vetch had previously opposed this view of the subject, by the statement that gonorrhœal ophthalmia occurs too frequently to admit of the probability of its correctness.

In support of the above-mentioned views, Dr. Vetch brings forward a case where the ophthalmic affection accompanying a gonorrhœa was accompanied also with an attack of rheumatism in other parts, and the ophthalmia, as it generally is in analogous cases, was *sclerotic*, not *conjunctival*, inflammation; whilst that produced by inoculation affects only the conjunctiva.

The inflammation of the sclerotica is very frequently, in the cases under especial consideration, communicated to the conjunctiva; and so is decidedly idiopathic inflammation of the sclerotica from other causes. It is but seldom, Dr. Vetch believes, that "the gonorrhœal action is translated to the conjunctiva, without attacking the sclerotic coat also." These facts strongly favour the author's opinion of the nature of gonorrhœal ophthalmia as it most ordinarily occurs; and several others, which our limits will not permit us to notice, are brought forward by him in its support.

The author says but little respecting the treatment of gonorrhœal sclerotic inflammation; but we may suppose that he does not consider that it should differ materially from sclerotic inflammation arising from other causes. There is, however, in this affection reason to fear a particularly rapid destruction of the cornea; and the warm poultices and fomentations recommended by most authors, are said by Dr. Vetch to be the most effectual means of assisting nature in this destruction. "Urged by the extreme necessity of the case," he adds, "I have been led to try a very free use of cold; and, from the trials I have made, I think it appears to be the best calculated to remove the disease. By the use of cold, I mean something more than the application of wet rags or compresses over the eye. The liquid applied should be cooled to a very low temperature, and renewed as soon as it ceases to be felt cold."

The subject of the last chapter of this work is the *purulent ophthalmia of infants*. The most remarkable point of diversity between this affection and the conjunctival ophthalmia of adults depends on the greater facility with which inflammation is transferred from the conjunctiva to the sclerotica in infants than in the latter subjects; and hence destruction of the cornea occurs in the former with much the greatest rapidity. The author mentions as its causes, more especially, some morbid secretion in the vagina of the mother, and an atmosphere infected with animal effluvia; and hence, that is from the latter cause, it is a disease of very frequent occurrence in lying-in hospitals, where the greatest attention is not paid to ventilation. Want of due cleanliness, and negligent exposure of the head to inordinate cold, seem also to be productive of it. Sometimes it ensues as a consequence of chronic inflammation of the glands of the eyelids. The disease, the author adds, seldom ceases in less than six weeks, and often continues from three to four months before the patient opens his eyes without assistance.

In the treatment, leeches will generally be advisable throughout the whole of the disease; and, "on the first accession of tumefaction, the best effect will often be experienced by the insertion of a small portion of ointment composed of lard, butter, or any animal fat, without wax, with a proportion of ten grains of the red nitrate of mercury to six drachms of the ointment. As the purulency advances, the liquor plumbi acetatis will be found no less serviceable than in other instances of purulent ophthalmia. It will be necessary, however, to ascertain that it has been boiled sufficiently to evaporate any free acid which might otherwise remain." When slough forms, a solution of nitrate of silver proves highly serviceable in assisting in its separation; and the recovery of the healthy state of the relaxed conjunctiva may be aided by the use of a solution of alum, or of the sulphate of copper, either dropped into the eye or injected with a syringe.

On revising the foregoing abstract, we have been somewhat perplexed in considering how we should act with it; for, although we have avoided, almost absolutely, producing any corollary observations or remarks, and have selected, almost exclusively, those parts of the work that are of a general character,—leaving a multitude of views subsidiary to them, as satellites are to their planets, quite unnoticed,—we find that it occupies an extent which is ill compatible with the numerous existing claims on the space of this Journal. But, those who agree with us respecting the character of the work will, probably, not wish that any thing we have here adduced from it had been omitted; and, in extending erasures further than we have done, we should charge ourselves with wilful injustice to the author, in consciously rendering the account we have attempted to give of his book less adequate to it than our abilities would permit.

A treatise of such a character as this cannot be expected to come frequently under our consideration. There is not another class of diseases than that which is the subject of it, that will, perhaps, admit of so much additional illustration: and we cannot venture even to hope that such an extensive series of important original observations will often be withheld from the public until they have been reflected on for twenty years,—by a man, too, to whom it is barely justice to assign the qualification of genius,—in an age like this, when every one who thinks he has got an original idea, hastens to swell it out to a great volume, which, like a bubble of soap-lather blown from the end of a tobacco-pipe, floats about for an instant; glitters with a few rays reflected from the little boys who stand round and gaze on it with admiration; then bursts, from its vacuity, when the vapour with which it was inflated has cooled; and disappears without leaving behind it even a vestige of its existence.

## Figures and Tables

**Figure f1:**